# Innovative Membrane Technologies for the Treatment of Wastewater Polluted with Heavy Metals: Perspective of the Potential of Electrodialysis, Membrane Distillation, and Forward Osmosis from a Bibliometric Analysis

**DOI:** 10.3390/membranes13040385

**Published:** 2023-03-28

**Authors:** Benjamín Carmona, Ricardo Abejón

**Affiliations:** Departamento de Ingeniería Química y Bioprocesos, Universidad de Santiago de Chile (USACH), Av. Libertador Bernardo O’Higgins 3363, Estación Central, Santiago 9170019, Chile

**Keywords:** heavy metals, pollution, wastewater treatment, electrodialysis, membrane distillation, forward osmosis, bibliometric analysis, research trends

## Abstract

A bibliometric analysis, using the Scopus database as a source, was carried out in order to study the scientific documents published up to 2021 regarding the use of electrodialysis, membrane distillation, and forward osmosis for the removal of heavy metals from wastewater. A total of 362 documents that fulfilled the search criteria were found, and the results from the corresponding analysis revealed that the number of documents greatly increased after the year 2010, although the first document was published in 1956. The exponential evolution of the scientific production related to these innovative membrane technologies confirmed an increasing interest from the scientific community. The most prolific country was Denmark, which contributed 19.3% of the published documents, followed by the two main current scientific superpowers: China and the USA (with 17.4% and 7.5% contributions, respectively). Environmental Science was the most common subject (55.0% of contributions), followed by Chemical Engineering (37.3% of contributions) and Chemistry (36.5% of contribution). The prevalence of electrodialysis over the other two technologies was clear in terms of relative frequency of the keywords. An analysis of the main hot topics identified the main advantages and drawbacks of each technology, and revealed that examples of their successful implementation beyond the lab scale are still scarce. Therefore, complete techno-economic evaluation of the treatment of wastewater polluted with heavy metals via these innovative membrane technologies must be encouraged.

## 1. Introduction

Due to its specific geographical, climatic, and productive characteristics, Chile is considered highly vulnerable to climate change, and one of the main consequences of climate change for Chile will be a lack of water. Chilean water resources are particularly sensitive because of the extreme harshness, frequency, duration, and significance of the “mega-drought” phenomenon affecting the country in the last years, which is expected to become a recurring and increasingly severe problem during the following decades, which will result in more severe arid conditions in vast regions of the country [1]. In fact, Chile has been clearly identified among the countries with the highest hydric stress, as shown in the data compiled in Table 1 [2].

The reduction of available water resources will force rationing them in a sustainable way in order to avoid additional anthropic incidence due to inadequate management and distribution [3]. Once these urgent needs have been recognized, the solutions to be proposed must consider the importance of avoiding the pollution of conventional water resources and using new unconventional sources of water, as well as considering the development of strategies and actions to manage them effectively [4,5]. Anthropogenic activities can adversely affect water quality by introducing contaminants and enhancing natural processes that reduce water quality. Such aspects can be especially important in semi-arid and arid zones, where population growth and the development of economic activities, such as agriculture and mining, stress the already-limited water supplies [6].

Mining, a critical productive sector in Chile, is increasingly associated with water risk, both in terms of limited water access for alternative uses and negative incidence on the quality of the surrounding water resources [7]. This is especially relevant where mines operate in water-scarce regions, or upstream of communities that rely on the same water source for consumption or agriculture. Water impacts are also increasingly at the center of social conflicts between local communities and mining companies. The exploitation of water resources for mining processes and related activities can result in returning lower-quality water to the environment, due to the presence of various minerals and the corresponding heavy metals [8]. Pollution of water resources by heavy metals implies very negative effects due to their high toxicity, high persistence, and rapid accumulation by living organisms [9]. The presence of some heavy metals, such as Cu, Cd, Zn, or Hg, in Chilean waters has been previously reported and well investigated [6,8,10,11,12].

Sustainable development goals (SDGs), the collection of 17 goals proposed by the United Nations in 2015, aimed to improve the world’s welfare by tackling social, environmental, and economic sustainability. Among SDGs, clean water and sanitation (SDG 6) and responsible consumption and production of resources (SDG 12) are directly related to the impacts of polluted water [13]. Taking into account the limited natural resources and the increasing pressure on ecosystems, one relevant strategy to minimize anthropogenic environmental impacts is to make better use of available water resources, including non-conventional resources.

Wastewater can be considered a non-conventional water resource, since its treatment and subsequent reuse provide a valuable way to rationalize the consumption of freshwater [14]. The regeneration of wastewater leads to a substantial increase in water supply for several purposes without any detrimental drawbacks, and is a solution to improve the vital ecological balance by simultaneously decreasing the uptake of freshwater from and the return of treated water to the environment. Consequently, water reclamation and reuse have increased in popularity. Reusing significant quantities of wastewater typically involves treatment of the wastewater in order to meet the water quality requirements of water-using activities [15]. However, the cost of wastewater treatment is directly related the concentration of pollutants and rises exponentially with increasing pollutant removal efficiency due to high initial contents. Therefore, the importance of assessing pollutant contents in the raw wastewater and the final treated water is undeniable in order to choose the most adequate treatment alternative [16].

Providing a proper wastewater treatment alternative for all types of pollutants is practically difficult and economically unaffordable, so the optimal solution for each particular case must be researched [17]. The selection of the optimal wastewater treatment process is more important in the case of reuse systems, in order to avoid any human health and environmental risks. A combination of biological, chemical, and physical removal processes are typical in wastewater treatment [18]. However, in the case of heavy metal removal, biological technologies are not adequate and the preferred processes are mainly based on physicochemical technologies. Physicochemical technologies for the removal of heavy metals from wastewater include chemical precipitation, ion exchange, adsorption, and membrane technologies [19].

Although membrane technologies present some disadvantages and technical challenges, they emerge as a desirable option for heavy metal removal from wastewater. Since the pioneering works related to membrane technologies in the 1970s, heavy metal removal from aqueous media has been one of their main applications, specifically in the case of pressure-driven membrane technologies such as ultrafiltration or reverse osmosis [20,21,22]. Among the main advantages of membrane technologies, the lack of auxiliary chemicals can be mentioned, while, quite often, a great percentage of the energy supplied in the separation process can be recovered from the treated water by different systems [23,24,25]. On the other hand, in most cases, in addition to the treated water stream, the application of membrane technologies results in the production of a concentrate stream with a higher metal concentration when compared to the raw wastewater, which must then be subject to additional treatment [26,27]. Nevertheless, if their respective limitations can be tackled and their performance further optimized, there is a very high possibility that membrane technologies will become the most versatile method for heavy metal removal [28].

In addition to traditional low-pressure (microfiltration and ultrafiltration) and high-pressure (nanofiltration and reverse osmosis) driven membrane technologies, other innovative technologies based on alternative gradients have appeared, such as osmotic pressure-driven membrane technologies (forward osmosis), thermally driven membrane technologies (membrane distillation), electrically driven membrane technologies (electrodialysis) and others (pervaporation, liquid membranes, etc.). The real potentiality of these technologies for the removal of heavy metals from wastewater is still only partially investigated [28]. Consequently, this work focused on a further-detailed analysis of three of them: electrodialysis, membrane distillation, and forward osmosis.

Electrodialysis is an electrically driven membrane technology that produces two streams with different solute concentrations flowing in alternate compartments, separated by ion exchange membranes [29]. These ion exchange membranes are selective membranes that allow the transport of ions when an electrical field is present. They can be categorized into anion exchange membranes and cation exchange membranes, which are selective to the permeation of anions and cations, respectively [30]. Electrodialysis can achieve high selectivity and product recovery without the employment of auxiliary chemicals, but its main limitation is the required electric potential, which can limit the economic viability of this technology. Moreover, the electrodialysis process can suffer membrane fouling caused by some inorganic and organic solutes. This fouling reduces the membrane’s performance and increases the corresponding energy consumption, so pretreatment is required in order to ensure an adequate inlet water quality.

Membrane distillation is an emerging thermally driven membrane technology based on the vapor pressure gradient across the surfaces of a porous hydrophobic membrane. The aqueous feed liquid in direct contact with the membrane cannot penetrate into the dry pores of the membrane, while volatile vapor molecules can permeate through the membrane. The permeated molecules can be collected or condensed by different methods [31]. This technology is widely used to concentrate aqueous solutions and provides a permeated distillate with high purity (because of the theoretical complete rejection of inorganic ions, macromolecules, and other non-volatile compounds), independently of the feed composition. In addition, it operates at relatively low temperatures, which allows it to take advantage of waste heat or the integration of renewable energy, such as solar power [32].

Forward osmosis is an osmotic pressure-driven membrane technology based on the flow of water through a semipermeable membrane from a diluted feed solution (low osmotic pressure) to a concentrated draw solution (high osmotic pressure) [33]. This process occurs naturally and does not require any external energy, since it is just opposite of the reverse osmosis process applied to water purification and desalination. The membrane fouling can be more easily controlled than in other membrane separation processes, and it is characterized by high water flux and strong adaptability. However, forward osmosis does not provide, as a result, direct purification; instead, the process leads to the dilution of the draw solution and the concentration of the feed solution [34].

The number of scientific documents related to water pollution and wastewater treatment that can be found in the bibliography is huge, so specific tools for a systematic management method to handle all of this information are required and bibliometric resources can provide a useful support. Bibliometrics, as defined by Pritchard, is the application of mathematical and statistical methods to books and other media of communication [35]. This research methodology is applied in library and information sciences to describe the distribution patterns of publications according to some given categories (such as topic, field, source, author, institution, or country) by means of quantitative analysis and statistical methods. It is a powerful tool for exploring, organizing, and analyzing large amounts of information in a quantitative manner. This approach enables access to relevant information and knowledge about the status of scientific research activities in specific disciplines, which helps researchers to identify novel schemes among the research [36]. Numerous bibliometric studies directly related to water pollution and wastewater treatment have been published [37,38,39,40,41,42,43,44,45,46,47,48,49,50,51,52].

The purpose of this work was to analyze, from a bibliometric perspective, the scientific literature published in Scopus related to the research on the application of electrodialysis, membrane distillation, and forward osmosis for the removal of heavy metals from wastewater. The documents found as a result of the corresponding bibliographic search were analyzed and evaluated according to several criteria (annual production, most prolific countries and institutions, main journals, and languages). Thereby, the quantitative features of the research were obtained and applied in order to identify the most important hot topics and research gaps in this field.

## 2. Data Sources and Methodology

The bibliographic search of the scientific literature related to electrodialysis, membrane distillation, and forward osmosis as technological options for wastewater treatment in order to remove heavy metals was based on the employment of Scopus. This database compiles abstracts and citations for academic journal articles and other scientific documents, covering over 84 million papers from over 7000 international publishers [53]. More than 27,000 scientific journals and 249,000 books are covered by Scopus, so taking into account these figures, Scopus can be considered the largest abstract and citation database of peer-reviewed literature. Scientific sources from all continents are considered, including non-English titles when abstracts in English are provided. Furthermore, more than half of the publications included in Scopus come from outside North America, with relevant production by European, Latin American, and Asian countries. As a result, Scopus offers an extensive range of peer-reviewed literature across the fields of science, technology, engineering, and mathematics (STEM).

The search within the Scopus database was completed in April 2022, with the choice of “*heavy metal*” AND (“*forward osmosis*” OR “*membrane distillation*” OR “*electrodialysis*”) as expressions used in the Article Title, Abstract, and/or Keywords fields of the search engine. Some keywords were introduced together with quotations to obtain only the papers that include both words in this exact sequence. This search was limited to works published until 2021, in order to discard those from 2022, as it can be only partially considered (it was not a completed year at the time of the search) and the comparison with previous years would not be fair. The total number of documents resulting from the search was 362.

The investigation of the scientific literature recovered after the systematic bibliographic search provided optimal conditions for attaining an improved understanding of the global research trends, which enables the discovery of past research gaps, the identification of present hot topics, and the proposal of future long-term research strategies. Therefore, the investigated aspects included in this bibliometric work did not only cover the quantitative description of the publications (such as annual production, most prolific countries and institutions, main journals, or most frequent languages), since it identified the most important research themes by the analysis of the most cited papers and the most frequent keywords.

## 3. Results and Discussion

### 3.1. Bibliometric Analysis of Research Trends on Innovative Membrane Technologies for Heavy Metal Removal (1956–2021)

#### 3.1.1. Publication Year, Document Type, and Language of Publications

**Figure 1:**
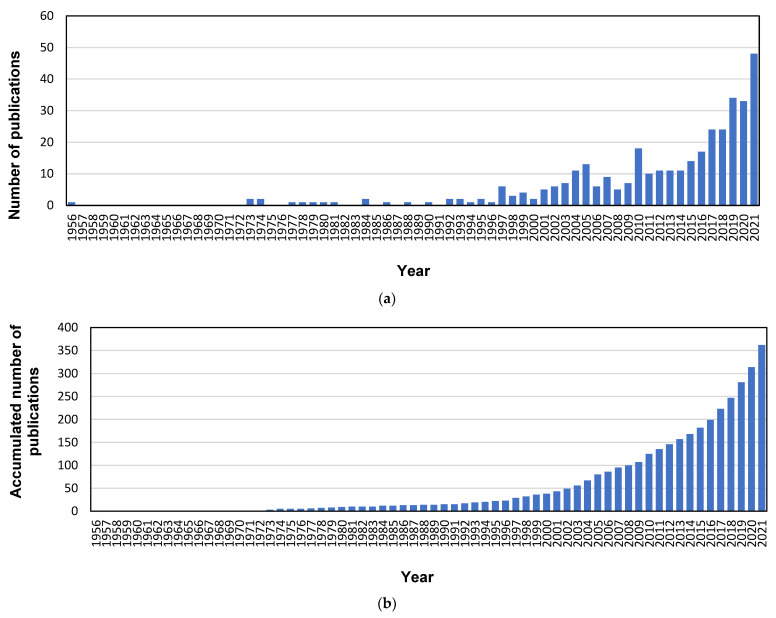
Annual (**a**) and accumulated (**b**) publication output.

The evolution of the annual scientific production in the sources of Scopus and the total number of accumulated documents over time are depicted in Figure 1. Three different stages can be distinguished. The first stage started in 1956, when the first document regarding electrodialysis and heavy metals was published [54], and it ended in 1996. Until that year, the annual scientific production regarding this field maintained very poor levels, with most years lacking any published documents, and the most productive years contributing only two documents. In fact, the accumulated production during this 41-year period comprised only 23 documents. This scarce production during the initial stage can be justified because electrodialysis, membrane distillation, and forward osmosis are considered innovative technologies, which deeply developed in the first years of the 21st century. The second stage ranged from 1997 to 2010, and followed a very irregular evolution in the annual production. This stage combined some specific years with high productions (for instance, the years 1997, 2004, 2005, and 2010 with 6, 11, 13, and 18 documents respectively), followed by periods that could not attain the peaks of production of these highly prolific years. This second stage can be considered the real dawn of these innovative technologies after the initial pioneering works published during the first stage. Lastly, the production during the third stage started with a constant plateau (10 or 11 documents per year during the 2011–2014 period), which was followed by an increasing trend in the last few years, from 14 documents in 2015 to 48 papers in 2021. This evolution demonstrated the high interest these technologies have gained in the last decade. Nonetheless, when the accumulated number of publications was analyzed, the corresponding evolution could be adjusted to an exponential growth, and the corresponding regression model was applied to the data. The obtained equation was y = 0.497·e^0.099·x^, where y represents the number of accumulated documents, and x represents the year (starting with one for the year 1956). The result was a good fitting, with a R^2^ value of 0.974, which confirmed that the selected innovative technologies (electrodialysis, membrane distillation, and forward osmosis) appeared as hot topics for the treatment of wastewater polluted with heavy metals, since the number of published papers regarding this topic has been continuously increasing in the last decade.

The different types of documents were evaluated, and seven types were identified. Articles constituted the most frequent document type, with 257 publications comprising 71.0% of total production, followed by reviews (46 documents that comprised 12.7% contribution) and conference papers (33 documents that comprised 9.1% contribution). The other less significant categories, which jointly constituted 7.2% of publications, include book chapters (16), conference reviews (7), books (2) and retracted work (1). The clear supremacy of articles when compared to other types of publications was totally consistent with the results mentioned by other authors who investigated the research published regarding water pollutants and treatment technologies [39,55,56]. However, the relatively high contribution of reviews is not so frequently reported, since the importance of the contributions in congress is significant in several engineering fields, including chemical, civil, and environmental [37,57,58,59], although some examples with a higher relative importance of reviews over congress contributions can be found [36,60,61]. Therefore, the higher importance of reviews when compared to conference papers can be linked to topics with elevated numbers of documents published, which can be revised and summarized by the corresponding reviews.

English was clearly the most frequently used language by the scientific community to publish documents related to the analyzed topic (95.3% of the documents were written in English). Several other languages were identified, with Chinese and Russian being the second and third most used languages, respectively (1.9% and 0.8% of the total number of papers, respectively). The remaining languages are compiled in Table 2, but they could be considered anecdotal, since each language only contributed with a single unique document. These results demonstrated, once again, that English has undoubtedly become the global lingua franca in the scientific research literature, specifically in engineering and the natural sciences, since more than 90% of the total publications were written in English [62,63,64].

#### 3.1.2. Publication Distribution of Countries and Institutions

The top 15 countries, those that published at least 9 documents, ranked by the total number of papers, are shown in Table 3. As the country affiliation is not an exclusive category (a document can be prepared by researchers from more than one country), documents can be linked to more than one country simultaneously due to international collaborations. Although typically, a reduced group of leading countries (including the USA and China as the two most relevant global leaders in scientific research) usually monopolizes international scientific production, in this case, the most productive country was Denmark, with 70 documents and 19.3% contribution. Despite the relatively high importance of research efforts promoted by Scandinavian countries, including Denmark, when their population is considered [38], the position of Denmark above China, which occupied the second position (63 documents and 17.4% contribution), and the USA, which occupied the third position (27 documents and 7.5% contribution), was surprising. Denmark is neither a global research superpower nor a country with severe water stress or water pollution, so the justification of its leading position in the ranking is not obvious. The joint contribution of these three first countries in the ranking accounts for around 45% of the total number of documents. Besides China, other Asian countries (such as India, Iran, South Korea, Malaysia, and Saudi Arabia) also appeared in the ranking. Two reasons may justify these contributions. On the one hand, some Asian countries are highly affected by hydric stress (for instance, Iran, Saudi Arabia, and India are included in Table 1), so the treatment of wastewater polluted with heavy metals seems a valid option for water recovery in these countries. On the other hand, water bodies severely polluted with heavy metals have been identified in some of these Asian countries [65,66,67,68,69], and efficient treatment options are required. Portugal appeared in the sixth position, before other European countries, including Spain, France, and the United Kingdom (10th, 13th, and 15th positions, respectively). South American contribution was only represented by Chile (in 11th position with 10 documents), which gave a clear idea about the efforts of this country in new technologies for heavy metals removal from wastewater.

The top 14 institutions, those with at least 5 documents, are compiled in Table 4. A Danish institution, the Technical University of Denmark, with 69 documents and 19.1% contribution, led the list. This university stands out for its engineering careers, as well as for being the most renowned university in Denmark, in addition to being in the top 100 ranking of universities worldwide on more than one occasion [70]. In the second position, a Portuguese university appeared (Universidade Nova Lisboa), which is specialized in the areas of engineering and natural sciences. However, its production was far below that attained by the Danish university, since only 16 documents were published (4.4% contribution). Among the top institutions in Table 4, five were in China and none in the USA. Therefore, although the USA was the third most productive country, this production was greatly shared among different institutions, and none of them published at least five documents. A Latin American institution, the Chilean university Universidad Técnica Federico Santa María, contributed as much as the leading Chinese institution (Chinese Academy of Sciences), both with eight documents.

#### 3.1.3. Distribution of Output in Subject Categories and Journals

The distribution of subject categories is represented in Table 5, which shows the 10 most popular categories (those with at least 13 documents). Once again, some papers can be included in more than one subject, since this is not an exclusive category. Consequently, the sum of the number of documents in each subject is higher than the total number of papers identified in the search. The ranking indicates that *Environmental Science* was the most common subject (199 documents, which represent 55.0% contribution), followed by *Chemical Engineering* (135 documents representing 37.3% contribution) and *Chemistry* (132 documents representing 36.5% contribution). Moreover, two other related subjects occupied the fourth and fifth positions: *Engineering* and *Materials Science*, with 93 and 65 documents, respectively. These results indicate three different approaches to this topic: Firstly, an environmental perspective related to the presence of heavy metals as pollutants in water samples must be highlighted. Secondly, the search for technically effective solutions with which to treat the wastewater polluted with heavy metals was the target of the engineering works, focused on the aforementioned innovative membrane technologies. Thirdly, the contributions to new materials and membranes and advanced knowledge of the mechanisms involved in these treatment options may explain the relevant roles of research works produced by chemists and materials scientists. These details provide strong evidence for the important interdisciplinary research efforts focused on the implementation of technical solutions based on innovative membrane technologies that can alleviate the problems derived from the presence of heavy metals in water.

The number of documents published in the most frequent journals is shown in Table 6. As an indicator of journal relevance, the corresponding impact factors (IFs) of the top 10 journals, those that published at least 7 articles, was included. Only the leading journal, *Journal of Hazardous Materials,* contributed more than 10% to the total scientific production (29 documents, which comprise 14.6% contribution). *Journal of Hazardous Materials* covers the hazards and risks that certain materials pose to public health and the environment, assessing the environmental impact (Environmental Science) and risk mitigation (Environmental Engineering) of hazardous materials. Two journals published 16 documents (8.0% contribution): *Chemical Engineering Journal* and *Desalination*. On the one hand, *Chemical Engineering Journal* focuses upon five aspects of chemical engineering: catalysis, chemical reaction engineering, environmental chemical engineering, green and sustainable science and engineering, and novel materials. On the other hand, *Desalination* is clearly focused on water desalting, including applications of desalination to seawater, groundwater, and waste waters by thermal, membrane, sorption, and hybrid processes. A quick overview of the topics covered by these three journals is enough to confirm, once again, the three interdisciplinary perspectives involved in the research in this field: environmental sciences, engineering and chemistry, and materials sciences. The relevance of these three journals leading the ranking is demonstrated by their IF values, all of them above 11. Another journal in the ranking, *Science of the Total Environment* (in the ninth position), also had an IF above 10. Nevertheless, the documents related to innovative membrane technologies for heavy metal removal have also been published in journals with lower relevance, such as *Desalination and Water Treatment* (IF = 1.72) and *Separation Science and Technology* (IF = 2.79).

#### 3.1.4. Most Frequently Cited Papers

The top 10 articles, ordered as a function of the number of citations they have received are presented in Table 7. The numbers of citations ranged from 190 for the least cited article to 5704 for the most popular article, which led the ranking. Although a more detailed analysis of the most relevant hot topics will be presented as a result of the study of the most frequently selected author keywords, an overview of the most cited publications gave an initial idea about some important aspects that have gained attention from researchers investigating these innovative membrane technologies.

The two most cited documents are generic reviews that cover the removal of heavy metals from wastewater [71,72]. In fact, the other two papers in the list, the ones that occupied the third and ninth positions, were directly related to the treatment options for removing heavy metals from a specific type of wastewater: produced water [73,74]. Produced water is a term used in the oil and gas industry to describe water that is produced as a byproduct during the extraction of oil and natural gas. This water is trapped in subsurface formations and is brought to the surface during the exploitation of the wells [75]. In addition to organic compounds related to hydrocarbons, produced water also contains high concentrations of heavy metals [76]. Other papers in Table 7 were focused on the removal of certain specific metals, such as copper [77,78], arsenic [79], or nickel [80]. The remaining two documents in the Top 10 list were not directly related to wastewater treatment: the one in the fourth position reviewed the surfactant-enhanced remediation of contaminated soils [81], while the one in the sixth position reviewed both the pollution by the electrochemical industry and the use of electrochemistry to clean water [82]. Surprisingly, none of the most cited documents were focused on membrane technologies for wastewater treatment.

#### 3.1.5. Distribution Analysis of Author Keywords and Trending Topics of the Research

**Figure 2:**
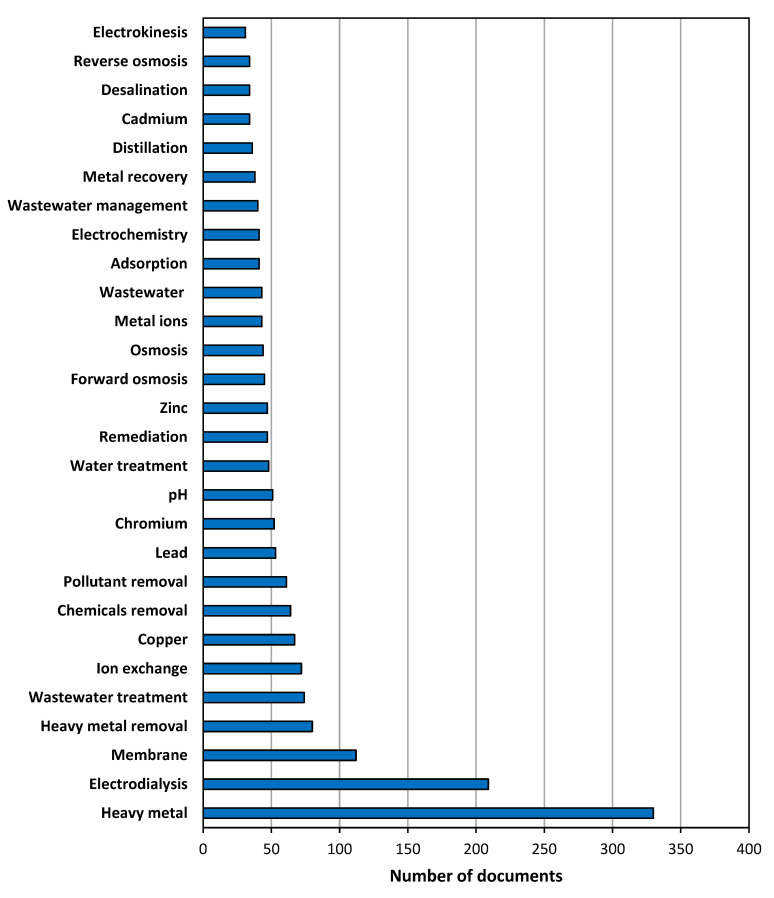
The top 29 most frequently used keywords (at least 30 results).

The list of the 29 most often used keywords (those that were mentioned at least 30 times) is shown in Figure 2. Obviously, “*heavy metal*” was the most frequent keyword, as it was selected in 330 articles. The next positions of the ranking corresponded to the other expressions introduced in the Article Title, Abstract, and Keywords fields of the search engine database: “*electrodialysis*” appeared 209 times, followed by “*heavy metal removal*” with 80 results. Indeed, these figures indicated that 91.2% of all the identified documents used “*heavy metal*” as a keyword, while this percentage was reduced to 57.7% for “*electrodialysis*”, the most mentioned technology among the three selected for analysis. After a further look at the terms listed, the identification of two other technologies revealed 45 results for “*forward osmosis*” (12.4% of the total number of documents) and 36 results for “*distillation*” (9.9% of the total number of documents), so the prevalence of electrodialysis over the other technologies was clear in terms of relative frequency. The appearance of “*membrane*” as a keyword in 112 results must be highlighted, and other expressions directly related to electrodialysis, e.g., “*ion exchange*”, appeared in the list with 72 times. Regarding other treatment options, “*adsorption*” and “*reverse osmosis*” also were included among the most frequent keywords, with 41 and 34 results, respectively. Keywords regarding the purpose of the treatments were frequent, since “*heavy metal removal*”, “*chemicals removal*”, and “*pollutant removal*” were included 80, 64, and 61 times, respectively, while “*wastewater treatment*”, “*water treatment*”, and “*remediation*” appeared 74, 48, and 47 times, respectively. Lastly, some examples of heavy metals appeared in Figure 2: “*copper*” (67 results), “*lead*” (53 results), “*chromium*” (52 results), “*zinc*” (47 results), and “*cadmium*” (34 results). This list of metals can provide a clear idea about the most investigated heavy metals, and one of them (copper) was previously identified as very relevant according to the analysis of the most cited documents.

### 3.2. Review of the Potential of Treatment Alternatives for Heavy Metal Removal from Wastewater

#### 3.2.1. Electrodialysis

Electrodialysis appeared as the most frequently referenced technological alternative among the three options considered in this work, with a great contribution by the researchers from Technical University of Denmark. A more detailed analysis revealed that most of the published works by this institution were focused on electrodialytic remediation [83]. Electrodialytic remediation differs from other electrokinetic remediation technologies in the use of ion exchange membranes for separation of solid waste and the operative liquid solutions. The solid waste is suspended in a central compartment separated from the electrolytes by ion exchange membranes. Between the anolyte and the solid suspension, an anion exchange membrane is placed, which hinders the passage of cations. Similarly, between the catholyte and the solid suspension, there is a cation exchange membrane, which hinders the transport of anions [84]. As a result, this membrane scheme forces the condition that the main direction of electromigration of the heavy metal ions is out from the suspension and into the electrode compartments. Electrodialytic remediation has been proposed as a fast and continuous in situ or off-site alternative for the treatment of several types of solid wastes contaminated with heavy metals, such as soils, sediments, ashes, mine tailings, air pollution control residues, and timber wastes [85,86].

The specific use of electrodialysis for the treatment of wastewater contaminated with heavy metals has gained attention in recent decades, especially since the 21st century started [87]. Several reviews that systematically compiled the most relevant works on electrodialysis applications in wastewater treatment for heavy metal removal have been published [88,89,90], including detailed analyses of more advanced configurations, such as bipolar membrane electrodialysis, displacement electrodialysis, or reverse electrodialysis, which have not been specifically covered in this work because they are not directly focused on the recovery of water. Although many industrial effluents can be successfully treated with electrodialysis, the wastewater produced in metal-finishing processes can be highlighted as the most illustrative example. These wastewaters contain high concentrations of heavy metals, such as copper, nickel, zinc, chromium, or silver [91,92,93], which can be recovered. In fact, the metal removal percentage from synthetic wastewater solutions via electrodialysis can obtain values above 99% when optimal conditions are applied [94], although the complex composition of real wastewater streams implied lower metal removal percentages [95]. The influence of the main process variables, such as applied voltage, membrane properties and feed flow rate, concentration, and temperature, has been deeply investigated [96]. The effect of several factors on the removal of heavy metals in an electrodialysis pilot plant was also studied [97]. The results of the experiment showed that increasing the linear flow velocity increased the voltage and the limiting current density. In addition, when the concentration of ions in the concentrate compartment increased, the ions back-diffused into the dilution compartment due to the concentration, so the ion concentration must be maintained at an appropriate level during the electrodialysis process to avoid these problems. As the number of cell pairs increased, because linear flow velocity and limiting current density increased, the removal efficiency of heavy metals also increased. Therefore, for highly concentrated wastewater, increasing the linear flow velocity, the applied voltage, and the number of cell pairs can effectively improve removal efficiency [98].

In some cases, the coupling of electrodialysis with other separation technologies has been evaluated. Electrowinning and chemical precipitation are suitable for both metal removal and the production of high-purity metal products, but require a high metal concentration in order to be effectively applied. Therefore, electrodialysis is a promising method for concentrating the metal species in wastewater [99]. This proposed sequential system was suitable for the treatment of copper plating wastewater, allowing for the recovery of the valuable metal. Electrodialysis has also been successfully coupled to separation processes based on supported liquid membranes [100]. Supported liquid membrane processes can recover valuable metals in the industrial wastewater. However, this separation is limited by the metal concentration gradient between the feed and stripping solutions. When the metal concentration in the stripping solution is low enough, transport of metal through the membrane can be accomplishment constantly. Taking this into consideration, electrodialysis can be placed after the liquid membrane, so the stripping solution was used as the feed solution for the electrodialysis process. This way, valuable metals can be adequately concentrated by electrodialysis, while the metal concentration in the stripping solution was maintained at low levels, which do not hinder ion transport through the liquid membrane.

Great research efforts have been applied to the development of new ion exchange membranes based on advanced materials and synthesis methodologies. Since these membranes are the integral part of the electrodialysis process, advances in this field can result in improved ion transport and selectivity under more favorable operational conditions. Very different approaches can be followed to produce new ion exchange membranes with improved performance, but most of them can be classified into the following categories: (a) introduction of organic and/or inorganic additives nanomaterials with special properties, such as hydrophilic, electrical charge, and absorptivity in the polymeric membrane matrixes; (b) use of a variety of ionic functional groups, such as phosphonic acid, sulfonic acid, and carboxylic acid in the matrix of the membranes; (c) blend of different types of polymers in the membrane fabrication to control the pores and the porosity of the membrane, and to change the mechanical strength and hydrophobicity of the matrix; and (d) surface modification by applying a thin modifier layer on the surface of the membranes [101].

The incorporation of different types of nanoparticles, such as zeolites or metal oxides, into the polymer matrix of ion exchange membranes can enhance their separation characteristics, and improve their thermal, mechanical, and chemical stability [102]. The particular case of magnetic nanoparticles can provide specific properties to ion exchange membranes that improve the removal of metal ions from wastewater. A new type of mixed matrix cation exchange membrane was prepared by incorporating magnetic cobalt ferrite nanoparticles to a PVC membrane for the removal of chromium ions via electrodialysis [103]. The membrane potential, charge density, permselectivity, ion exchange capacity, and mass flux were enhanced when the nanoparticles were incorporated, which could be linked to the strong affinity of the magnetic nanoparticles to heavy metal ion adsorption. Incorporation of inorganic clay mineral layers (such as montmorillonite or synthetic mica) has been demonstrated as a valid option to improve the mechanical properties of porous membrane supports. For example, the performance of polysulfone (PSf) and montmorillonite composite membranes for electrodialysis was analyzed [104]. The presence of advanced modified montmorillonite influenced the water contact angle and the pore size of the membrane. The rejection of zinc ions and the mass flow increased in the modified membranes, especially for the membranes that incorporated the functionalized form of the filler, which favored its dispersion in the matrix. Metal organic frameworks (MOFs) are another type of inorganic fillers successfully applied to the modification of ion exchange membranes for electrodialysis. A cation-selective MOF, such as UIO-66, provided cation selectivity to a polyvinylidene fluoride (PVDF) membrane with cation selectivity that enabled the successful application of the membrane in an electrodialysis system to remove lithium ions [105]. The consideration of membranes based on natural biopolymers, such as chitosan or cellulose, can provide many advantages (low cost, wide availability, and/or biodegradability). Ion exchange membranes made of chitosan with silver particle content have been tested for removing iron ions from synthetic wastewater [106]. The electrical characteristics of the biopolymeric membrane enriched with silver ions highlighted the suitability of the system for removing iron ions. In fact, the value of current efficiency was higher for the biopolymeric membrane in comparison to other polymeric membranes. New ion exchange membranes have been synthetized via a layer-by-layer coating method through chemical crosslinking. The interactions between the layers correspond to different types of bonds, such as hydrogen, ionic, and covalent bonds. Researchers evaluated the coating of a chitosan-co-activated carbon nanoparticle layer on a PVC-based cation exchange membrane [107]. Enhanced adherence between layers was obtained using glutaraldehyde as a crosslinking reagent. The prepared membrane showed a smoother and more hydrophilic surface compared to the pristine PVC membrane, which promoted the effective regeneration of the fouled membrane using ultrasonic waves, and the electrodialysis experiments demonstrated a good ability for the removal of heavy metal ions, including copper, nickel, and lead. The hydrogels can be employed as modifier agents on the surface of ion exchange membranes to improve the ionic interactions and control the transport of ions. When hydrogels are present in an aqueous environment, their functional groups may be changed into negatively charged groups, enabling interactions with cations. Therefore, hydrogels are able to modify the performance of cation exchange membranes. Hydrogels based on polysaccharides, polyvinyl alcohol, and modified cellulose have been widely used. The incorporation of 2-acrylamido-2-methylpropane sulfonic-acid-based hydrogel (AMAH) to a polyvinylchloride (PVC) cation exchange membrane improved the ionic permeability and ionic flux of the membrane, while its electrical resistance was reduced [108].

The potential widespread use of electrodialysis for treating wastewater with heavy metals may be hindered by membrane scaling problems. Several studies have investigated the influence of the pH on the removal of heavy metals via electrodialysis and, while the removal of some ions was pH independent, other metals (such as lithium, calcium, magnesium, strontium, zinc, uranium, or selenium) were highly affected by the pH conditions [109]. In fact, at high pH levels, the precipitation of insoluble substances and their interaction with other impurities resulted in membrane deposition in the form of scaling. This, in turn, had a negative impact on the system’s effectiveness, as it caused an increase in stack resistance and a reduction in the removal of total dissolved solids. For instance, the scale produced on the surface of the ion exchange membranes when treating copper model wastewater solutions was identified as a mixture of Cu(OH)_2_ and CuO, most likely formed as a consequence of the reaction of water hydrolysis products (H^+^ and OH^−^) and Cu^2+^ ions [110]. This study revealed that the anion exchange membranes were more prone to scaling than cation exchange membranes. Nevertheless, this scaling resulted in lower overall ion removal efficiency, which required a higher voltage to drive ion transport through the membranes. Moreover, the cation transport mechanism through scaled membranes was clearly more unstable and heterogeneous than through a membrane without scaling. A detailed analysis of the precipitated calcium and magnesium scaling on ion exchange membranes when liquid digestates were treated by electrodialysis identified different scalants depending on the type of membrane and the feed solutions [111]. Observed scalants on cation exchange membranes were vaterite, amorphous calcium carbonate, and struvite, while amorphous calcium carbonate was not found on anion exchange membranes. When synthetic solutions were treated instead of real digestates, calcite appeared as the main scalant. The membranes with higher selectivity for divalent ions resulted in a faster reduction in the electric current, causing more serious membrane scaling.

Preliminary economic evaluation results revealed that electrodialysis can be combined with alternative processes to treat wastewater with high concentrations of heavy metals under cost-competitive conditions. For instance, the wastewater obtained after the washing of soils polluted with Fe, Cd, and Zn was subject to treatment by chemical precipitation, bipolar membrane electrodialysis, and activated carbon adsorption [112]. The result of the corresponding cost analysis indicated that the combined process could be regarded as a highly efficient, cost-effective, and feasible technology with which to treat soil washing wastewater. It should be noted that this economic evaluation did not consider any extra advantages (such as the reuse of acids and alkali, as well as the recovery of heavy metals), which could make it more economically preferable and promote its further implementation. Other studies have demonstrated that the recovery of these resources from wastewaters can provide remarkable economic profits and compensate for the operation costs [113].

#### 3.2.2. Membrane Distillation

Membrane distillation is a thermal process that enables the transport of only vapor molecules through the pores of a hydrophobic membrane, while the aqueous liquid feed solution cannot penetrate into the dry pores of the membrane. Therefore, liquid/vapor interfaces are formed at the entrances of the membrane pores. In order to maintain the pressure difference between both surfaces of the membrane, which acts as driving gradient, four traditional configurations can be selected: direct contact membrane distillation, air gap membrane distillation, vacuum membrane distillation, and sweeping gas membrane distillation, this last option being the least frequent for the treatment of wastewaters polluted with heavy metals [114,115,116]. Percentages of heavy metal rejection between 95 and above 99.9% have been frequently reported for membrane distillation processes, with permeated water fluxes in the interval from 4 to 50 L/h·m^2^ [117].

Several research studies have investigated deeply, by means of both theoretical and experimental investigations, the effects of the membrane characteristics (such as membrane nature, thickness, pore size, etc.) and the operating parameters (such as feed and coolant temperatures or feed flow rate and composition) of the different configurations of membrane distillation [118]. These efforts have derived the development of modeling and simulation tools that predict the performance of the newly developed membranes, modules and processes [119,120,121].

The two main drawbacks of membrane distillation through long-term continuous operation are pore wetting and fouling [122]. On the one hand, the membrane surface can be susceptible to interaction with the species present in the feed solution and increase its hydrophilicity. Consequently, the liquid phase can wet and penetrate into the pores of the membrane and reduce its separation efficiency dramatically. On the other hand, these interactions with the membrane surface can increase the possibility of fouling due to the accumulation of unwanted compounds which might clog the pores and consequently decrease the permeate flux. Therefore, great research efforts have been applied in order to solve these problems through the development of more adequate membranes for membrane distillation [123].

The synthesis of superhydrophobic membranes that minimize the problems caused by pore wetting have been deeply investigated. The hydrophobic/hydrophilic nature of a membrane highly depends on the membrane preparation materials and methods. Hydrophobic and very inert polymers such as polytetrafluoroethylene (PTFE) and polyvinylidene fluoride (PVDF) can be highlighted as the most frequent materials for the synthesis of membranes to be employed in membrane distillation, replacing traditional polypropylene (PP) and polyethylene (PE) membranes [124,125]. Further functionalization of the membrane through the addition of nanoparticles (in most cases inorganic materials) contributes to the production of an improved superhydrophobic surface due to the presence of a set of functional groups at the surface of the membrane. Metal oxides, such as silica, alumina, titania, or graphene-derived materials, are among the preferred materials to be added in order to obtain improved characteristics [126,127,128,129,130]. A composite PVDF membrane with functionalized alumina (Al_2_O_3_) using isostearyl acids showed a higher water contact angle (150° vs. 132° when alumina was not present), which resulted in increased permeate flux (5.9% increase) and very high metal rejection, with a rejection percentage above 99.3% [131]. The introduction of a Ti-O-Ti condensed structure into a PVDF electrospun membrane was proposed using titanium tetra isopropoxide as an additive, subject to subsequent post-treatment by hydroxylation and deprotonation [116]. Although the developed membrane exhibited better performance in terms of long-term stability and heavy metal rejection, the incorporation of the titania did not improve the permeate flux of the membrane. Recently, novel hydrophobic polymers have also been proposed to replace the traditional formulations based on PVDF and PTFE. For instance, styrene-butadiene-styrene (SBS) electrospun membranes have been prepared [132]. The SBS membranes exhibited excellent hydrophobicity characteristics, with a contact angle higher than 130° and adequate permeate flux rate (54.4 L/h·m^2^). Moreover, the addition of alumina nanoparticles enhanced the performance of the SBS membranes substantially in terms of permeate flux and heavy metal retention, due to higher porosity and hydrophobicity.

Accumulation and interaction of both organic and inorganic species with the membranes employed in membrane distillation can cause significant fouling and reduce the permeate flux. When this technology is applied to the treatment of wastewater with a high content of heavy metals, inorganic salts precipitate and form scaling on the membrane surface [133]. Characterization of the fouling layers formed during membrane distillation revealed the occurrence of membrane scaling that was mainly due to the deposition of precipitated metal oxides, hydroxides, oxyhydroxide, carbonates, and sulphates [115]. In fact, the corresponding flux reductions caused by these fouling layers were highly dependent on the concentration of metals and inorganic anions, as well as the presence of bulk organics in feed water. Nevertheless, the fouling can be effectively mitigated, achieving conditions very similar to the initial ones, by means of periodic cleaning of the membranes with adequate rinsing formulations, such as dilute acid solutions [134,135]. Another option for controlling the fouling is the implementation of pretreatments that can reduce the content of the most problematic species. As an illustrative example, synthetic acid rock drainage was subject to a chemical-free thermal precipitation pretreatment before being fed to the membrane distillation process [136]. In this case, membrane scale formation was greatly reduced and the permeate flux was not significantly reduced.

Some researchers have analyzed the synergies of coupling membrane distillation with other technologies to recover the heavy metals present in the wastewater. Membrane distillation extracts reusable water from the feed solution while producing a more concentrated retentate solution, with higher metal concentrations. If enough freshwater can permeate the membrane, the retentate solution will achieve concentration values close to saturation. Under these circumstances, the concentrated solution can be cooled and sent to crystallization [137]. Even the highly concentrated metal (zinc and nickel) values attained when the membrane distillation retentate is sent to crystallization did not have a particular effect on the rejection of the membrane process. Adsorption is another alternative for coupling with membrane distillation. The concentrated metal solutions that result from the membrane distillation process imply a more favorable scenario to apply adsorption (higher equilibrium concentrations and faster loading of the adsorbent) [138,139]. Different adsorbents, such as zeolites or silica-based materials, have been tested and successful results were obtained, since the metal concentration increased by more than 2.5 times while recovering 80% of the water for reuse purposes. Nevertheless, Fe and Al precipitates from a concentrated solution deposited onto the hollow fiber membrane caused its hydrophobicity to be dramatically reduced, causing partial wetting of the pores. To avoid this problem, previous heavy metal removal by zeolite, specifically Fe and Al, mitigated membrane fouling on the surface of the hollow fiber membrane, so a configuration with two stages of adsorption (the first one as pretreatment before the membrane distillation and the second one as post-treatment after it) has been suggested.

The energetic aspects of membrane distillation are very relevant. The temperature plays a vital role in the performance of the process. The optimal operational conditions related to the temperatures have been clearly identified: maximal feed inlet temperature and minimal coolant inlet temperature maximize the performance of the membrane distillation process [140]. The implementation of these optimal thermal conditions is limited by the economic costs of the heating and cooling requirements. Consequently, the energetic and economic aspects of membrane distillation must be considered simultaneously during the design of the processes. The energy consumption required by different membranes (characterized by different pore sizes) within a wide range of concentrations of different metals in the feed solution was monitored and the results revealed that it was almost independent of membrane pore size, metal type, and feed concentration [114]. The researchers proposed an optimal energy requirement in the interval between 16 and 22 kWh/kg permeate. Membrane distillation can be easily operated with sustainable and efficient thermal energy sources, such as waste heat [141,142,143] or solar energy [144,145]. Solar-driven membrane distillation considering specific module design, which promoted flash vaporization, achieved high permeate fluxes with complete removal of arsenic and avoided pore wetting despite prolonged use of the membrane module [146]. More advanced membrane distillation configurations proposed a new approach that eliminates the need to heat all feed water, as solar heat is localized only on the surface of the membrane [147]. This type of process relies upon the membrane material, which must be a highly solar-absorbing photothermal material. Activated carbon particles at different concentrations were used in the prepared PVDF membranes as components for solar energy recovery and obtained effective photothermal membranes. While the results showed that the presence of activated carbon particles as an additive in the PVDF significantly improved solar absorption (97%), porosity (60.6%), and thermal stability, the corresponding water contact angles were reduced and the risk of pore wetting increased. Nevertheless, complete techno-economic analysis, with a specific focus on energy, of the implementation of membrane distillation processes for water recovery from heavy-metal-loaded wastewaters are still required.

#### 3.2.3. Forward Osmosis

The forward osmosis process has obtained renewed interest nowadays and it has demonstrated that a very high retention of heavy metals can be achieved. Percentages of rejection between 93 and above 99.8% have been more frequently reported, achieving water fluxes through the membrane in the range 10–50 L/h·m^2^ [28,117]. Nevertheless, the number of scientific studies conducted on heavy metal removal using forward osmosis is still far away from the number of studies focused on desalination.

One of the most significant critical challenges is the need for specific membranes designed for forward osmosis to replace the use of membranes designed for other purposes (such as nanofiltration or reverse osmosis) and overcome the main technical drawbacks, such as membrane fouling. Both organic and inorganic contaminants can block membrane pores and cause severe fouling of the membranes employed in forward osmosis, therefore decreasing the mass transfer and water flux [148]. An adequate pretreatment of the feed solution can significantly reduce the fouling, but great research efforts have been focused on the synthesis of improved membranes with better antifouling properties. Complete reviews that cover the synthesis and modifications of the different layers of forward osmosis membranes, including chemical modifications of the bulk membrane or the specific membrane surfaces, and the incorporation of nanomaterials or chemical additives, have been published [149,150].

The modification of the structure of the active layer of forward osmosis membranes can be achieved through the addition of chemicals that modify the formulation and structure of the active layer or through incorporation of an additional coating. Examples of both approaches have been published. On the one hand, a novel thin film composite forward osmosis membrane was prepared using a glass nanofiber supporting layer and a swellable active layer of bovine serum albumin and polyamide [151]. The addition of this chemical provided swellable sites and improved the rejection of heavy metal ions. In addition, the increased porosity and pore size of the glass nanofiber supporting layer provided smooth flow channels, accelerating the flow process and alleviating the concentration polarization. On the other hand, the effects of the addition of a polydopamine coating over the active layer of a conventional thin film composite forward osmosis membrane on the mass transport and fouling behavior were studied [152]. The coated membrane reduced the internal concentration polarization inside the membrane, leading to an enhanced water flux, and presented an improved antifouling performance compared to the control membrane. However, when the coating duration lasted excessively (longer than 2 h), reduced water flux resulted due to the significantly increased hydraulic resistance of the coated membranes.

Recent studies have shown that additives can decrease the membrane fouling by increasing its hydrophobicity. The incorporation of nanoparticles and nanotubes has been validated as an effective way to improve membrane performance. Graphene oxide is capable of absorbing heavy metals, owing to its functional groups and high specific surface area, which can enhance the membrane stability and antifouling properties [153]. In fact, when compared with traditional TFC membranes, composed of a polyamide active layer over a polysulfone support layer, the membranes with incorporated graphene oxide exhibited higher hydrophilicity, porosity, water permeability, and salt rejection, as well as lower reverse solute flux and internal concentration polarization. Recently, metal-organic frameworks (MOFs) have been grown on graphene oxide in order to take advantage of the properties provided by MOFs and overcome their instability [154]. The developed nanocomposite membrane exhibited improved stability, as well as higher water permeation and heavy metal removal performance. The nanofunctionalization of membrane support layers with titania nanotubes and magnetite oxide hybrid nanoparticles was also investigated [155]. In this case, the enhanced performance of the membrane, with a higher water flux, but without losing its heavy metal rejection capacity, was mainly attributed to the improved substrate hydrophilicity, which effectively reduced the internal polarization concentration. Another study proposed the consideration of an interlayer formed by nanoparticles between the support and active layers [156]. Polydopamine nanoparticles were selected and an increasing amount of them resulted in a denser and thicker selective layer, which implied decreased water and solute permeability. Other approaches have investigated the addition of different materials, such as zeolites [157]. Composite zeolite hollow fiber membranes, with UV-curable resin as a secondary coating material, were synthetized. The preparation of the membrane started by depositing a zeolite membrane onto alumina hollow fiber, followed by a photopolymerization process once the outer layer was fully covered with a perfluorochemical solution. Although the hydrophobicity of the membrane was enhanced, the coating layer of resin did not improve the stability of the membrane as heavy metals interacted with the fluorine atoms of the resin and caused agglomeration of the zeolite particles and subsequent membrane defects.

The charge of a membrane surface affects rejection, fouling, and flux performance during the forward osmosis process, since the intrinsic electronegative properties of polymeric forward membranes can cause membrane fouling when treating wastewater-containing positively charged contaminants (such as heavy metal cations) because of the interactions via electrostatic attraction. Therefore, the synthesis of novel positively charged membranes emerges as a useful solution to reduce the fouling. Positively charged membranes via complexation under mild conditions without the use of organic solvents or corrosive chemicals have been prepared. For example, the simple complexation of polyamide membranes by introducing either single Fe^3+^ or Fe^3+^ and betaine improved the membrane permeability (which was at least doubled), while enhanced antifouling properties appeared as demonstrated when exposed to cetylpyridinium chloride solutions [158]. Biomimetic aquaporin membranes have demonstrated comparable water flux, higher contaminant rejection, improved antifouling properties, and lower reverse solute flux when compared to conventional membranes [159].

In addition to improved membrane designs, based on advanced materials and modifications, some researchers have investigated the application of membrane vibration as a method to reduce fouling and improve the performance of the process [160]. In fact, moderate vibration was able to increase the water flux substantially due to enhanced promoted flow velocity, as well as the improvement of the effective osmotic pressure caused by the reduction of the solute buildup [161].

Reverse solute transport is characterized by relevant amounts of draw solutes permeating from the draw side of the membrane. This phenomenon not only implies a loss of ions from the draw solution, but also contaminates the feed solution. Moreover, a further problem can occur: the reaction of draw solutes with species present in the feed solution. This was the case of a work that studied the treatment of acid mine drainage (with the presence of heavy metals such as Cu, Co, Cr, Fe, Mn, or Zn) by forward osmosis using NH_4_HCO_3_ as draw solute [162]. The permeation of ammonia and bicarbonate ions into the feed solution resulted in the appearance of a precipitate over the feed side of the membrane. These drawbacks require the selection of better draw solutes, which can enhance water permeation and avoid reverse solute transport. Although simple inorganic salts, such as MgCl_2_, can achieve a relatively low reverse solute flux [163], more sophisticated draw solutes have been investigated.

Three-dimensional multi-charge metallic complexes have been proposed as suitable draw solutes. These chemicals produce extremely high osmotic pressure due to the formation of multi-ionic species in the water, but also minimize reverse solute flux because of their structures [164]. Examples of these draw solutes include a cobalt hydroacid complex, Na_4_[Co(C_6_H_4_O_7_)_2_] 2H_2_O [165]; a chromium hydroacid complex in a tetramethylammonium salt, [N(CH_3_)_4_]_3_[Cr(C_2_O_4_)_3_] [164]; or betaine-based polynuclear complexes based on copper, nickel and zinc [166]. Another approach that has been successfully implemented is the addition of a complexing agent, which can interact with the draw solute. For instance, the addition of poly(sodium 4-styrenesulfonate) to a MgCl_2_ draw solution reduced the reverse solute flux due to the complexation and the increased molecular weight of the new complex [167].

Nevertheless, the application of forward osmosis for the treatment of wastewater polluted with heavy metals is mostly investigated in lab-scale experiments only. The upscaling of the process to evaluate the performance on pilot-scale or even real full-scale tests must be promoted [168]. Within this framework, the evaluation of long-term fouling behavior, membrane cleaning methods, and operational conditions will be more relevant. In addition, forward osmosis cannot be considered a stand-alone process for water recovery, since the permeated flux dilutes the draw solution. Additional stages are required for treating the draw solution in order to recover or eliminate the draw solute and obtain reusable water. Several technologies to be coupled with forward osmosis, including other membrane technologies, have been previously proposed as promising and effective options for treating the diluted draw solutions [169]. Nevertheless, complete economic evaluation, including the energetic aspects, should be carried out in order to promote the implementation of forward osmosis.

## 4. Conclusions

A bibliometric analysis was conducted in order to prepare an overview of the research related to the use of electrodialysis, membrane distillation, and forward osmosis in treating wastewater contaminated with heavy metals (information was gathered about annual publications, document types, languages, countries, institutions, categories, journals, and keywords) using the Scopus database. Although the number of accumulated publications about this topic followed an exponential evolution during the complete time range, three different periods have been identified, including an initial phase with very scarce production from 1956 to 1996, followed by a very irregular evolution phase until 2010, characterized by peaks and valleys. After this year, the production experienced a significant increase, achieving the maximal annual production in 2021 (48 documents). Denmark, surprisingly, was the most productive country by total number of publications (19.3% contribution to the total number of documents), followed by a couple of the current main scientific superpowers (China and the USA, with 17.4% and 7.5% of contributions, respectively). The results revealed that Environmental Science was the most common subject, followed by Chemical Engineering, Chemistry, Engineering, and Materials Science. Three different approaches to the research related to these innovative membrane technologies could be identified: the first was an environmental perspective related to the presence of heavy metals as pollutants in water samples; the second considered the search of technically effective processes for the treatment of wastewater contaminated with heavy metals by engineering solutions; and the third looked for new materials and membranes and advanced knowledge of the mechanisms involved in these treatment options from the perspective of chemists and materials scientists. The analysis of the most frequent keywords showed that electrodialysis was the most mentioned technology, appearing in 57.7% of the total number of documents. The other two technologies were not as frequently selected as keywords: 45 results for forward osmosis (12.4% of the total number of documents) and 36 results for distillation (9.9% of the total number of documents).

Although electrodialysis was the most frequently referenced technology, with a great contribution by researchers from Technical University of Denmark, a deeper analysis showed that most of the published works by this institution were focused on electrodialytic remediation, which is focused on the treatment of solid wastes instead of wastewater. Relevant research efforts have been applied to the development of new ion exchange membranes for electrodialysis, considering the incorporation of nanoparticles and inorganic fillers, the replacement of traditional polymers by biopolymers or the use of hydrogels. The coupling of electrodialysis with other treatment technologies, such as chemical precipitation or supported liquid membranes, has proven to be a successful option. The application of electrodialysis to the treatment of wastewater produced in metal-finishing processes has been investigated in detail and the influence of the main operation variables on the process performance has been characterized. Nevertheless, the long-term performance of electrodialysis is threatened by fouling and scaling, so effective solutions to this problem must be proposed. In addition, the optimization of the energy consumption of wastewater treatment processes based on electrodialysis is still pending.

The effects of the membrane characteristics (membrane composition, thickness, pore size, etc.) and the operating parameters (feed and coolant temperatures, feed and coolant flow rate, and feed composition) of the different configurations of membrane distillation have been investigated in detail. The synergies of coupling membrane distillation with other technologies, such as crystallization or adsorption, which can enable the recovery of the heavy metals removed from water, have been investigated. New superhydrophobic membranes, which can prevent pore wetting (one of the most important drawbacks of membrane distillation), have been synthetized. However, as in the case of electrodialysis, membrane fouling must be avoided in order to warrant the effective long-term performance of the membrane distillation processes. Regarding energy consumption, examples that employed residual heat or direct solar energy have been presented.

Forward osmosis can be considered as the least mature technology among the three evaluated in this work. Several problems related to this technology must be solved before it could be considered as a valid option for the treatment of wastewater polluted with heavy metals. Firstly, the development of new membranes specifically designed for application in forward osmosis is still pending. Secondly, the selection of the most adequate draw solute and its concentration in the draw solution is not an easy task. Thirdly, reverse solute transport must be impeded in order to avoid the presence of the draw solute in the treated stream. Fourthly, scaling and fouling problems caused by the wastewater must be managed. Finally, if the recovery of the treated wastewater is desired, forward osmosis mandatorily requires the design of a subsequent stage for removal of the draw solute, which implies additional costs to the wastewater treatment.

More research, not only at a lab-scale experimental level but also at a scaled-up industrial level, is still needed in order to advance in the implementation of the three innovative membrane technologies covered in this work. The selection of optimal membranes and definition of optimal operational conditions depending on the characteristics of the wastewater to be treated (metals present, their concentrations, presence of other chemicals, feed flowrate, etc.) will contribute to the design of effective processes for the removal of heavy metals. More detailed techno-economic evaluation of the treatment processes based on electrodialysis, membrane distillation, and forward osmosis can significantly pave the way to their implementation.

## Figures and Tables

**Table 1:** Ranking of the top 20 countries with the highest hydric stress, according to the water risk index (WRI).

Ranking	Country	WRI	Ranking	Country	WRI
1	Qatar	4.97	11	San Marino	4.14
2	Israel	4.82	12	Bahrain	4.13
3	Lebanon	4.82	13	India	4.12
4	Iran	4.57	14	Pakistan	4.05
5	Jordan	4.56	15	Turkmenistan	4.04
6	Libya	4.55	16	Oman	4.04
7	Kuwait	4.43	17	Botswana	4.02
8	Saudi Arabia	4.35	18	Chile	3.98
9	Eritrea	4.33	19	Cyprus	3.97
10	UAE	4.26	20	Yemen	3.97

**Table 2:** The languages employed by the publications.

Language	Publications	Contribution (%)
English	345	95.30
Chinese	7	1.93
Russian	3	0.83
Japanese	1	0.28
Lithuanian	1	0.28
Moldavian	1	0.28
Persian	1	0.28
Romanian	1	0.28
Undefined	2	0.55

**Table 3:** The top 15 most productive countries (at least 9 documents).

Country	Publications	Contribution (%)
Denmark	70	19.34
China	63	17.40
USA	27	7.46
India	23	6.35
Iran	21	5.80
Portugal	20	5.52
Australia	18	4.97
South Korea	14	3.87
Malaysia	12	3.31
Spain	12	3.31
Chile	10	2.76
Canada	9	2.49
France	9	2.49
Saudi Arabia	9	2.49
United Kingdom	9	2.49

**Table 4:** The top 14 most productive institutions (at least 5 documents).

Institution	Publications	Contribution (%)
Technical University of Denmark (Denmark)	69	19.06
Universidade Nova Lisboa (Portugal)	16	4.42
Universiti Tekknologi Malaysia (Malaysia)	9	2.49
University of Technology Sydney (Australia)	9	2.49
Universidad Técnica Federico Santa María (Chile)	8	2.21
Chinese Academy of Sciences (China)	8	2.21
Arak University (Iran)	7	1.93
Donghua University (China)	7	1.93
Shenzhen University (China)	6	1.66
KU Leuven (Belgium)	6	1.66
Chinese Ministry of Education (China)	5	1.38
Silesian University of Technology (Poland)	5	1.38
Fuzhou University (China)	5	1.38
Escola Superior Agraria de Coimbra (Portugal)	5	1.38

**Table 5:** The top 10 most popular subject categories (at least 13 documents).

Ranking	Subject	Publications	Contribution (%)
1	Environmental Science	199	54.97
2	Chemical Engineering	135	37.29
3	Chemistry	132	36.46
4	Engineering	93	25.69
5	Materials Science	65	17.96
6	Biochemistry, Genetics and Molecular Biology	32	8.84
7	Energy	21	5.80
8	Earth and Planetary Sciences	16	4.42
9	Physics and Astronomy	14	3.87
10	Medicine	13	3.59

**Table 6:** The top 10 most popular journals (at least 7 documents).

Source	2021 IF	Publications	Contribution (%)
Journal of Hazardous Materials	14.22	29	14.57
Chemical Engineering Journal	13.27	16	8.04
Desalination	11.21	16	8.04
Journal of Membrane Science	8.74	12	6.03
Desalination and Water Treatment	1.72	11	5.53
Electrochimica Acta	7.34	11	5.53
Chemosphere	8.94	10	5.03
Separation Science and Technology	2.79	10	5.03
Science of the Total Environment	10.75	9	4.52
Waste Management	8.81	7	3.52

**Table 7:** The top 10 most cited papers.

Ranking	Articles	Times Cited
1	Title: **Removal of heavy metal ions from wastewaters: A review** Authors: Fu, F., Wang, Q. Source: *Journal of Environmental Management* Published: 2011	5704
2	Title: **New trends in removing heavy metals from industrial wastewater** Authors: Barakat, M.A. Source: *Arabian Journal of Chemistry* Published: 2011	1773
3	Title: **Review of technologies for oil and gas produced water treatment** Authors: Fakhru’l-Razi, A., Pendashteh, A., Abdullah, L.C., (...), Abidin, Z.Z. Source: *Journal of Hazardous Materials* Published: 2009	1421
4	Title: **Surfactant-enhanced remediation of contaminated soil: A review** Authors: Mulligan, C.N, Yong, R.N, Gibbs, B. F Source: *Engineering Geology* Published: 2001	807
5	Title: **Waste biomass adsorbents for copper removal from industrial wastewater: A review** Authors: Bilal, M., Shah, J.A., Ashfaq, T., Haroon, H., (...), Mahmood, Q. Source: *Journal of Hazardous Materials* Published: 2013	367
6	Title: **Electrochemistry: As cause and cure in water pollution—An overview** Authors: Vasudevan, S., Oturan, M.A. Source: *Environmental Chemistry Letters* Published: 2014	255
7	Title: **Possible treatments for arsenic removal in Latin American waters for human consumption** Authors: Litter, M.I., Morgada, M.E., Bundschuh, J. Source: *Environmental Pollution* Published: 2010	233
8	Title: **Copper removal from industrial wastewater: A comprehensive review** Authors: Al-Saydeh, S.A., El-Naas, M.H., Zaidi, S.J. Source: *Journal of Industrial and Engineering Chemistry* Published: 2017	227
9	Title: **State of the art of produced water treatment** Authors: Jiménez, S., Micó, M.M., Arnaldos, M., Medina, F., Contreras, S. Source: *Chemosphere* Published: 2018	210
10	Title: **Nickel recovery/removal from industrial wastes: A review** Authors: Coman, V., Robotin, B., Ilea, P. Source: *Resources, Conservation and Recycling* Published: 2013	190

## Data Availability

The authors can provide the employed data on demand.
